# Characterization of the Proteostasis Roles of Glycerol Accumulation, Protein Degradation and Protein Synthesis during Osmotic Stress in *C. elegans*

**DOI:** 10.1371/journal.pone.0034153

**Published:** 2012-03-28

**Authors:** Kristopher Burkewitz, Keith P. Choe, Elaine Choung-Hee Lee, Andrew Deonarine, Kevin Strange

**Affiliations:** 1 Boylan Center for Cellular and Molecular Physiology, Mount Desert Island Biological Laboratory, Salisbury Cove, Maine, United States of America; 2 Department of Biology, University of Florida, Gainesville, Florida, United States of America; Consejo Superior de Investigaciones Cientificas, Spain

## Abstract

Exposure of *C. elegans* to hypertonic stress-induced water loss causes rapid and widespread cellular protein damage. Survival in hypertonic environments depends critically on the ability of worm cells to detect and degrade misfolded and aggregated proteins. Acclimation of *C. elegans* to mild hypertonic stress suppresses protein damage and increases survival under more extreme hypertonic conditions. Suppression of protein damage in acclimated worms could be due to 1) accumulation of the chemical chaperone glycerol, 2) upregulation of protein degradation activity, and/or 3) increases in molecular chaperoning capacity of the cell. Glycerol and other chemical chaperones are widely thought to protect proteins from hypertonicity-induced damage. However, protein damage is unaffected by gene mutations that inhibit glycerol accumulation or that cause dramatic constitutive elevation of glycerol levels. Pharmacological or RNAi inhibition of proteasome and lyosome function and measurements of cellular protein degradation activity demonstrated that upregulation of protein degradation mechanisms plays no role in acclimation. Thus, changes in molecular chaperone capacity must be responsible for suppressing protein damage in acclimated worms. Transcriptional changes in chaperone expression have not been detected in *C. elegans* exposed to hypertonic stress. However, acclimation to mild hypertonicity inhibits protein synthesis 50–70%, which is expected to increase chaperone availability for coping with damage to existing proteins. Consistent with this idea, we found that RNAi silencing of essential translational components or acute exposure to cycloheximide results in a 50–80% suppression of hypertonicity-induced aggregation of polyglutamine-YFP (Q35::YFP). Dietary changes that increase protein production also increase Q35::YFP aggregation 70–180%. Our results demonstrate directly for the first time that inhibition of protein translation protects extant proteins from damage brought about by an environmental stressor, demonstrate important differences in aging- versus stress-induced protein damage, and challenge the widely held view that chemical chaperones are accumulated during hypertonic stress to protect protein structure/function.

## Introduction

Maintenance of the conformation, concentration, interactions, localization, and hence function of cytoplasmic proteins is termed protein homeostasis or “proteostasis”. Proteostasis is maintained by the tightly integrated activities of gene transcription, RNA metabolism and protein synthesis, folding, assembly, trafficking, disassembly and degradation [1], [2]. Proteostasis mechanisms are highly conserved across evolutionarily divergent species and are essential for life.

Hypertonicity-induced cellular water loss and associated cell shrinkage increase cytoplasmic ionic strength and macromolecular crowding. Elevated ionic strength can denature proteins while macromolecular crowding increases protein-protein interactions that can lead to protein aggregation [3]–[5]. While it is widely believed that hypertonic stress damages cytoplasmic proteins in vivo, there is little direct evidence to support this idea.

We demonstrated recently in the genetic model organism *C. elegans* that hypertonic stress causes aggregation and misfolding of diverse fluorescently tagged foreign and endogenous proteins and proteins with temperature sensitive point mutations [6], [7]. Protein damage is rapid. Aggregation of a polyglutamine YFP reporter is observable in muscle cells with <1 h of hypertonic stress and aggregate volume doubles approximately every 10 min. Aggregate formation is irreversible and triggered by events that occur after as little as 10 min of exposure to hypertonic conditions. Numerous endogenous native proteins also undergo striking and rapid aggregation during hypertonic stress [6].

Survival of *C. elegans* in hypertonic environments requires the function of genes that play essential and conserved roles in the destruction of damaged proteins. Acclimation of worms to mild hypertonic stress suppresses protein damage that normally occurs under more extreme conditions [6], [7]. Our combined studies thus demonstrate that detection, repair, removal and suppression of protein damage are critical factors that define the ability of cells and organisms to survive in osmotically stressful environments. The speed at which protein damage occurs in *C. elegans* during hypertonic stress and the relative ease of measuring this damage provides a unique model for defining the mechanisms utilized by eukaryotic cells to maintain proteoastasis during environmental insults.

The suppression of protein damage we observed in worms acclimated to mild hypertonic stress [6], [7] could be due to three physiological processes: 1) accumulation of organic osmolytes, which are believed to function as chemical chaperones [3], [8], [9], 2) increases in protein degradation activity [7], and/or 3) increases in the overall molecular chaperoning capacity of the cell [10]. The current studies were carried out to identify which of these processes protect the cellular proteome from damage during hypertonic stress. Surprisingly, our studies failed to identify a protein protective role for glycerol, which is the major organic osmolyte accumulated by *C. elegans* under hypertonic conditions [11], [12]. Upregulation of protein degradation activity also played no role in the acclimation process. Instead, we find that an increase in the overall molecular chaperoning capacity of cells plays the predominant role in reducing protein damage. At least part of the increase in chaperone capacity can be attributed to acute reductions in protein synthesis, which reduces the load on the chaperone network. Our studies provide important new insights into the mechanisms utilized by cells and organisms to cope with protein damaging environmental stressors, demonstrate important differences in aging- versus stress-induced protein damage, and raise interesting and provocative questions about the physiological roles of organic osmolytes.

## Results

Proteins that contain contiguous repeats of glutamine residues aggregate spontaneously and have been used to identify genes, cellular processes, and physiological conditions that influence protein folding and aggregation in several experimental models including *C. elegans* (e.g. [13]–[15]). A transgene containing 35 contiguous glutamine residues fused to yellow fluorescent protein (Q35::YFP) is soluble and uniformly distributed throughout muscle cells until adult *C. elegans* are 3 days old, after which the protein aggregates spontaneously with age [15]. Q35::YFP begins to aggregate in 1-day old adult worms within 30–60 min after exposure to hypertonic stress [6], [7].

**Figure 1:**
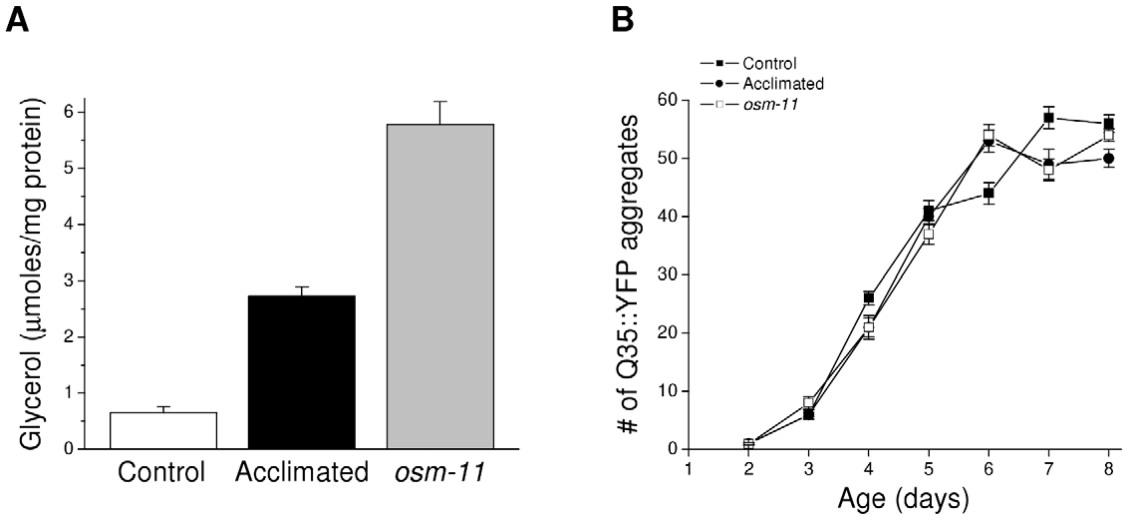
Effect of elevated glycerol levels on aging-induced aggregation of Q35::YFP. *A:* Whole worm glycerol levels in controls worms, worms acclimated to 200 mM NaCl and *osm-11* mutant animals. (*n* = 4 samples of ∼4000 worms/sample). *B:* Time course of aging-induced Q35::YFP aggregation in control, acclimated and *osm-11* mutant animals. (*n* = 7 experiments with 10–15 worms/experiment).

*C. elegans* accumulates the organic osmolyte glycerol when exposed to hypertonic stress [11]. Organic osmolytes function as chemical chaperones to minimize some types of protein damage [3], [8], [9]. To test the role of glycerol in suppressing protein damage, we quantified spontaneous, aging-induced aggregation of Q35::YFP in worms grown on agar containing 51 or 200 mM NaCl. We also examined spontaneous Q35::YFP aggregation in worms carrying a loss-of-function allele of *osm-11*. OSM-11 is a secreted protein that functions in Notch signaling and may play a role in modulating behavioral responses to environmental stress [16], [17]. Loss of OSM-11 activity constitutively activates *gpdh-1* expression and glycerol accumulation [12], [18]. As shown in Figure 1A, acclimation to 200 mM NaCl or loss of *osm-11* function increase whole worm glycerol levels ∼4- and ∼10-fold, respectively. However, despite large increases in glycerol levels, spontaneous aggregation of Q35::YFP was not suppressed in acclimated and *osm-11* mutant worms compared to control animals (Figure 1B).

**Figure 2:**
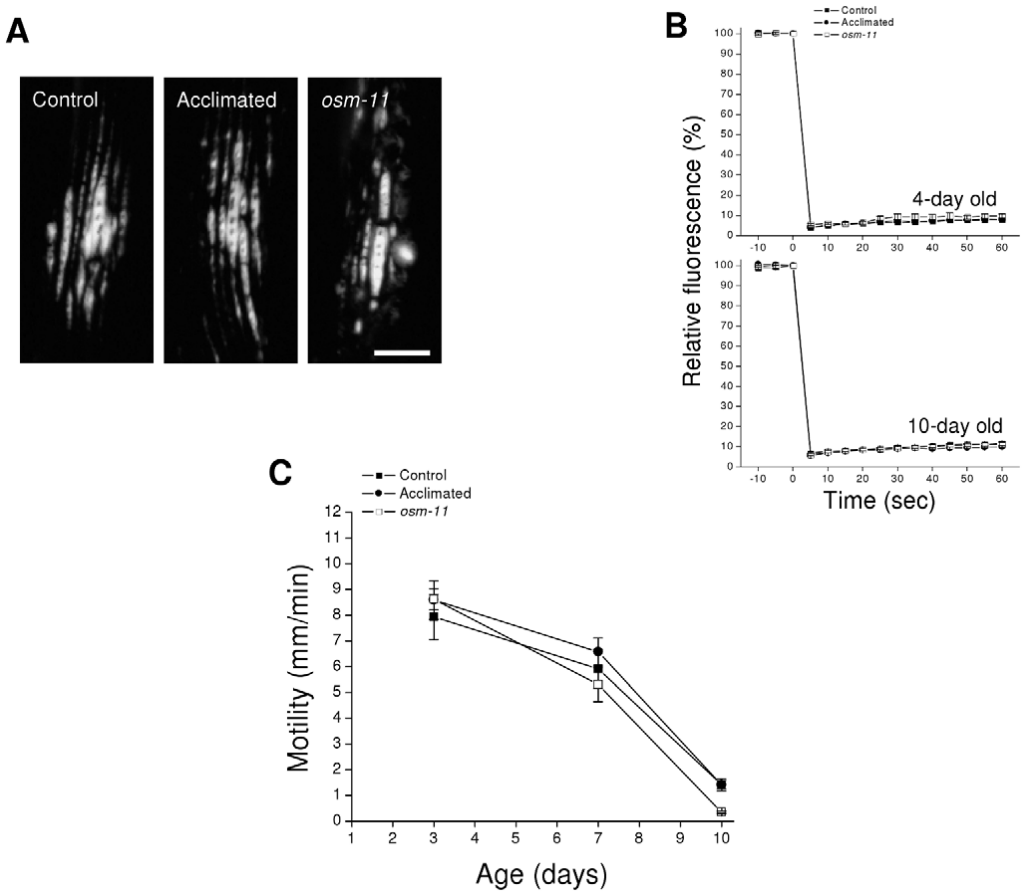
Effect of elevated glycerol levels on the properties of age-induced Q35::YFP aggregates. *A:* Fluorescence micrographs of aggregate morphology in body wall muscle cells of control worms, worms acclimated to 200 mM NaCl and *osm-11* mutant animals. Images were taken from 7-day old adult worms. Scale bar is 10 µm. *B:* Time course of bleaching and fluorescence recovery in aggregates of young (4-day old) and old (10-day old) adult control, acclimated and *osm-11* worms (*n* = 3). *C:* Aggregate toxicity in control, acclimated and *osm-11* worms. Toxicity is measured as reductions in motility, which is mediated by body wall muscle cells. (*n* = 5–12).

To further characterize possible effects of glycerol on Q35::YFP aggregation, we examined age-induced aggregate morphology and solubility. *C. elegans* body wall muscle cells comprise a contractile myofilament lattice and a noncontractile cell body that contains the cytoplasm and cell organelles. Q35::YFP aggregates were localized by confocal microscopy. Elongated, dense aggregates that aligned with myofilaments were localized to the contractile lattice (Figure 2A). We also observed more diffuse aggregates in the cell body that frequently colocalized with nuclei (not shown). This morphology and pattern of localization is similar to that described previously [19]. No obvious differences in morphology and localization were detected in control, 200 mM NaCl acclimated and *osm-11* mutant worms.

We used fluorescence recovery after photobleaching (FRAP) analysis as described previously [7] to assess the solubility of Q35::YFP present in aggregates. FRAP analysis was carried out on aggregates in young (4-day old) and old (10-day old) adult worms. As shown in Figure 2B, FRAP was undetectable in punctate YFP structures observed in young and old control, 200 mM NaCl acclimated and *osm-11* mutant worms. These results demonstrate 1) that Q35::YFP is localized to aggregates where individual proteins are immobile, and 2) that the presence of high levels of glycerol has no effect on Q35::YFP solubility.

Finally, we examined Q35::YFP toxicity. Q35::YFP aggregates damage muscle cells and cause gradual paralysis [15]. It is conceivable that glycerol minimizes aggregate toxicity. As shown in Figure 2C, the age associated decline in motility brought about by Q35::YFP aggregation was similar in control, acclimated and *osm-11* mutant worms.

**Figure 3:**
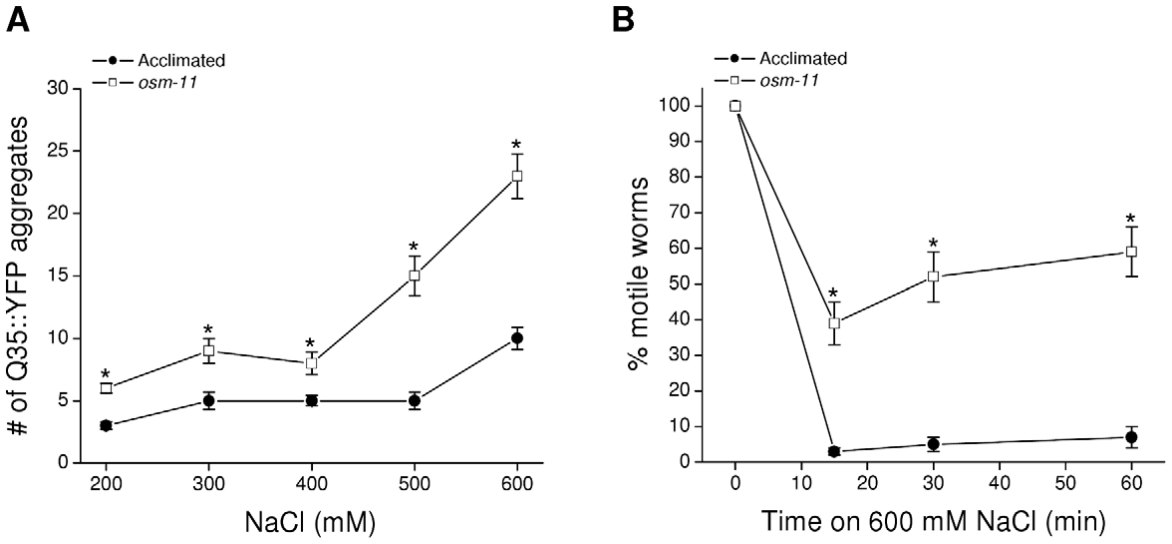
Effect of elevated glycerol levels on hypertonic stress-induced aggregation of Q35::YFP. *A:* Aggregation of Q35::YFP measured after a 24 h exposure of *osm-11* or acclimated worms to media containing 200–600 mM NaCl. (*n* = 13–15). *P<0.01 compared to acclimated animals. *B:* Time course of motility changes in *osm-11* and acclimated worms exposed to 600 mM NaCl. Acclimated worms become paralyzed within 10–15 min due to water loss. *osm-11* mutants lose less water due to elevated glycerol levels (see Figure 1A) and therefore remain motile compared to acclimated worms. (*n* = 13–21). *P<0.0001 compared to acclimated animals.

Data in Figures 1 and 2 indicate that high levels of glycerol do not suppress aging-induced Q35::YFP aggregation or alter aggregate properties. However, the ability of acclimation to 200 mM NaCl to suppress hypertonicity-induced Q35::YFP aggregation, but not spontaneous aggregate formation (Figure 1B) suggests that the two aggregation processes are fundamentally different. To directly assess the possible role of glycerol in suppressing hypertonic stress-induced Q35::YFP aggregation, we exposed *osm-11* worms to high NaCl concentrations. As shown in Figure 3A, *osm-11* worms exhibit significantly (P<0.01) greater Q35::YFP aggregation at all NaCl concentrations tested (i.e., 200–600 mM) compared to acclimated animals.

Cell shrinkage and increased macromolecular crowding almost certainly play a role in driving Q35::YFP aggregation in hypertonically stressed worms [7]. A trivial explanation for the results shown in Figure 3A is that *osm-11* animals experience more extreme shrinkage compared to acclimated animals. To test this possibility, we quantified motility in acclimated and *osm-11* worms exposed acutely to 600 mM NaCl. Motility in *C. elegans* requires internal hydrostatic pressure in order for the outer cuticle to function as an exoskeleton that body wall muscles pull against for motility. Loss of body water and hydrostatic pressure causes temporary paralysis until fluid balance is restored. As shown in Figure 3B, virtually all worms acclimated to 200 mM NaCl remain paralyzed for at least 60 min when exposed to 600 mM NaCl. In contrast, ∼60% of the *osm-11* worms remain motile under the same conditions. This result is consistent with the observation that glycerol levels in *osm-11* worms are ∼2.5 times higher than those in animals acclimated to 200 mM NaCl (Figure 1A). Higher internal solute concentrations and resulting osmotic pressure reduce water loss in hypertonic environments. Thus, even though *osm-11* worms have higher glycerol levels and lose less water during hypertonic stress, Q35::YFP aggregation is more extensive than that observed in acclimated worms. Taken together with data shown in Figure 1B, these results demonstrate 1) that glycerol plays no role in suppressing either spontaneous or hypertonic stress-induced Q35::YFP aggregation, and 2) that acclimation to 200 mM NaCl activates non-osmolyte mechanisms that prevent Q35::YFP aggregation in response to more extreme hypertonic stress.

In addition to transgenic reporter proteins, endogenous proteins aggregate when *C. elegans* is exposed to hypertonic stress. Aggregated proteins are present in the detergent insoluble fraction of whole worm protein extracts [6]. To determine whether glycerol levels influence endogenous protein aggregation, we utilized *osm-11* mutants and *gpdh-1; gpdh-2* double mutant worms. GPDH-1 and GPDH-2 are both required for hypertonicity-induced glycerol accumulation. Glycerol accumulation in mutant worms carrying deletion mutations in both enzymes (i.e., *gpdh-1; gpdh-2*) is reduced ∼60% when they are exposed to 200 mM NaCl [12].

**Figure 4:**
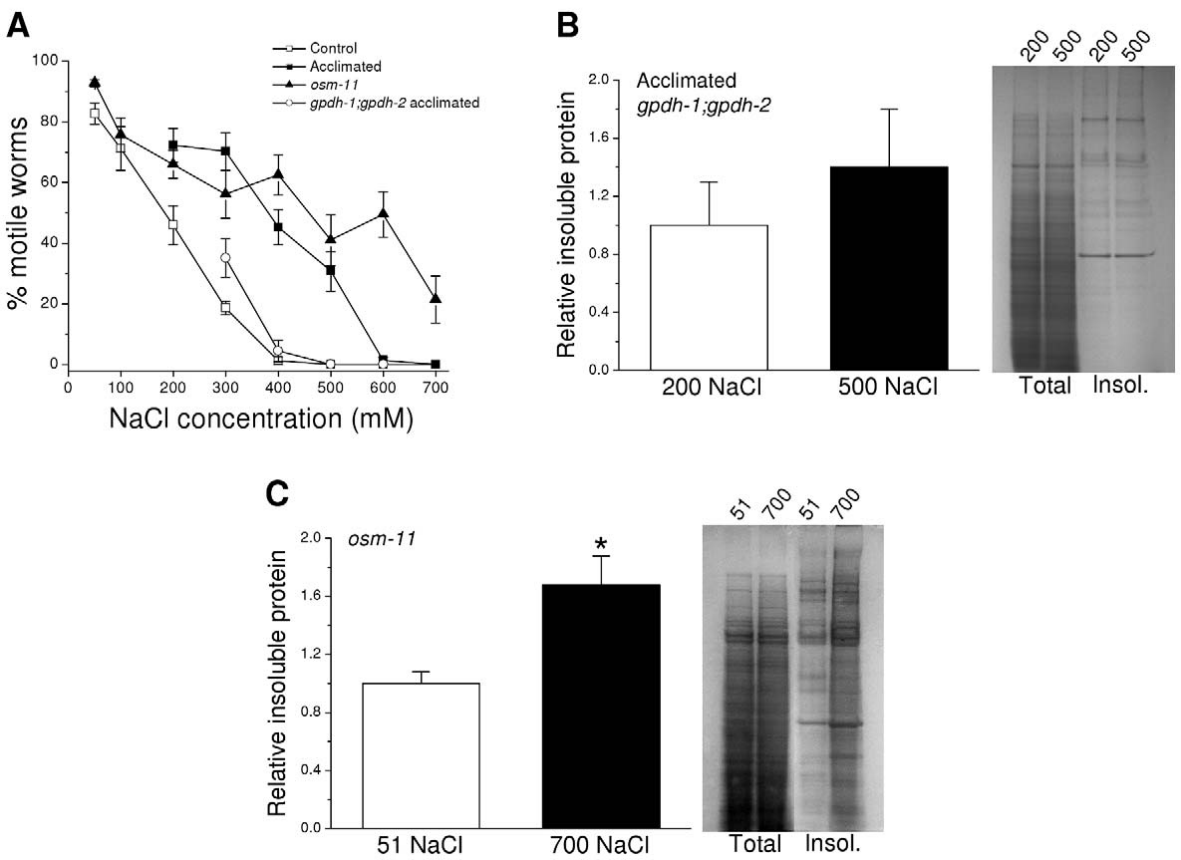
Effect of elevated glycerol levels on hypertonic stress-induced aggregation of endogenous proteins. *A:* Effect of increasing NaCl concentrations on motility in control, acclimated, *osm-11* and acclimated *gpdh-1; gpdh-2* worms. *gpdh-1; gpdh-2* mutants lack functional GPDH-1 and GPDH-2 enzymes resulting in greatly reduced glycerol accumulation under hypertonic stress conditions [12]. (*n* = 5–18 experiments with 15–60 worms/experiment). *B:* Left panel, relative insoluble protein in acclimated *gpdh-1; gpdh-2* worms maintained in either 200 mM NaCl or exposed to 500 mM NaCl for 4 h. Insoluble protein was quantified as a fraction of total protein and is plotted relative to that observed in worms maintained on 200 mM NaCl. (*n* = 3 experiments with 4000–5000 worms/experiment). Right panel, examples of SDS-PAGE gels of total and detergent insoluble (insol.) proteins isolated from acclimated *gpdh-1; gpdh-2* worms maintained in 200 mM NaCl or exposed to 500 mM NaCl. *C:* Left panel, relative insoluble protein in *osm-11* worms grown under control conditions (51 mM NaCl) or exposed to 700 mM NaCl for 4 h. Insoluble protein was quantified and plotted in the same manner as described in *B*. (*n* = 3 samples of 4000–5000 worms/sample). *P<0.03 compared to animals maintained on 51 mM NaCl. Right panel, examples of SDS-PAGE gels of total and detergent insoluble (insol.) proteins isolated from *osm-11* worms exposed to 51 or 700 mM NaCl.

We first carried out acute motility studies in the two mutant strains and in wild type worms exposed to a range of NaCl concentrations in order to assess how changes in glycerol accumulation impact whole animal water loss. As expected, wild type worms acclimated to 200 mM NaCl and *osm-11* mutants showed much higher levels of motility over a range of NaCl concentrations compared to unacclimated wild type animals (Figure 4A). *gpdh-1; gpdh-2* double mutant worms acclimated to 200 mM NaCl exhibited reductions in motility similar to those observed of unacclimated worms (Figure 4A). The higher levels of motility in *osm-11* mutants and acclimated wild type worms are consistent with reduced water loss due to elevated glycerol levels. In contrast, suppression of glycerol accumulation in the *gpdh-1; gpdh-2* double mutants results in increased water loss and reductions in motility similar to those of wild type worms that have not been exposed to hypertonic stress.

As we have shown previously [6], acclimation of wild type worms to 200 mM NaCl suppresses aggregation of endogenous proteins when animals are exposed to more extreme hypertonic stress. To assess whether glycerol plays a role in reducing aggregation, we acclimated *gpdh-1; gpdh-2* double mutants to 200 mM NaCl and then isolated detergent insoluble proteins from them before (i.e., control) and 4 h after exposure to 500 mM NaCl. As shown in Figure 4B, exposure of acclimated *gpdh-1; gpdh-2* mutants to 500 mM NaCl had no significant (P>0.4) effect on the level of aggregated endogenous proteins despite extensive water loss (Figure 4A). Exposure of unacclimated wild type worms to 500 mM NaCl in contrast increases endogenous protein aggregation 220% [6], [7]. Thus, acclimation inhibits protein aggregation independently of GPDH function and glycerol accumulation.

Endogenous proteins undergo substantial aggregation when unacclimated wild type worms are exposed to hypertonic stress [6]. To further assess the possible role of glycerol in suppressing protein aggregation, we isolated detergent insoluble protein fractions from *osm-11* worms maintained under normal growth conditions and *osm-11* mutant worms exposed to 700 mM NaCl for 4 h. As shown in Figure 4C, 700 mM NaCl caused a significant (P<0.03) increase in aggregated endogenous protein levels. This increase is similar to that observed in unacclimated wild type worms exposed to 400 or 500 mM NaCl [6] even though water loss is considerably lower in *osm-11* mutants (Figure 4A). Data in Figures 4B–C taken together with our previous findings, suggest strongly that glycerol plays little or no role in suppressing endogenous protein aggregation and that mechanisms other than glycerol accumulation must inhibit aggregation in acclimated worms.

Organic osmolytes are widely thought to function in the prevention and reversal of protein misfolding (e.g., [20], [21]). While there is extensive in vitro evidence to support this idea, few studies have characterized the chemical chaperone roles of organic osmolytes in vivo. We examined the role of glycerol in minimizing hypertonic stress-induced protein misfolding using worm strains harboring temperature sensitive (ts) alleles of *let-60* and *unc-15*, which encode a ras GTPase and paramyosin, respectively. At low or permissive temperatures, ts mutations are thought to have little impact on protein folding and associated function. However, at elevated temperatures, these mutations cause protein misfolding that gives rise to dysfunction and mutant phenotypes.

**Figure 5:**
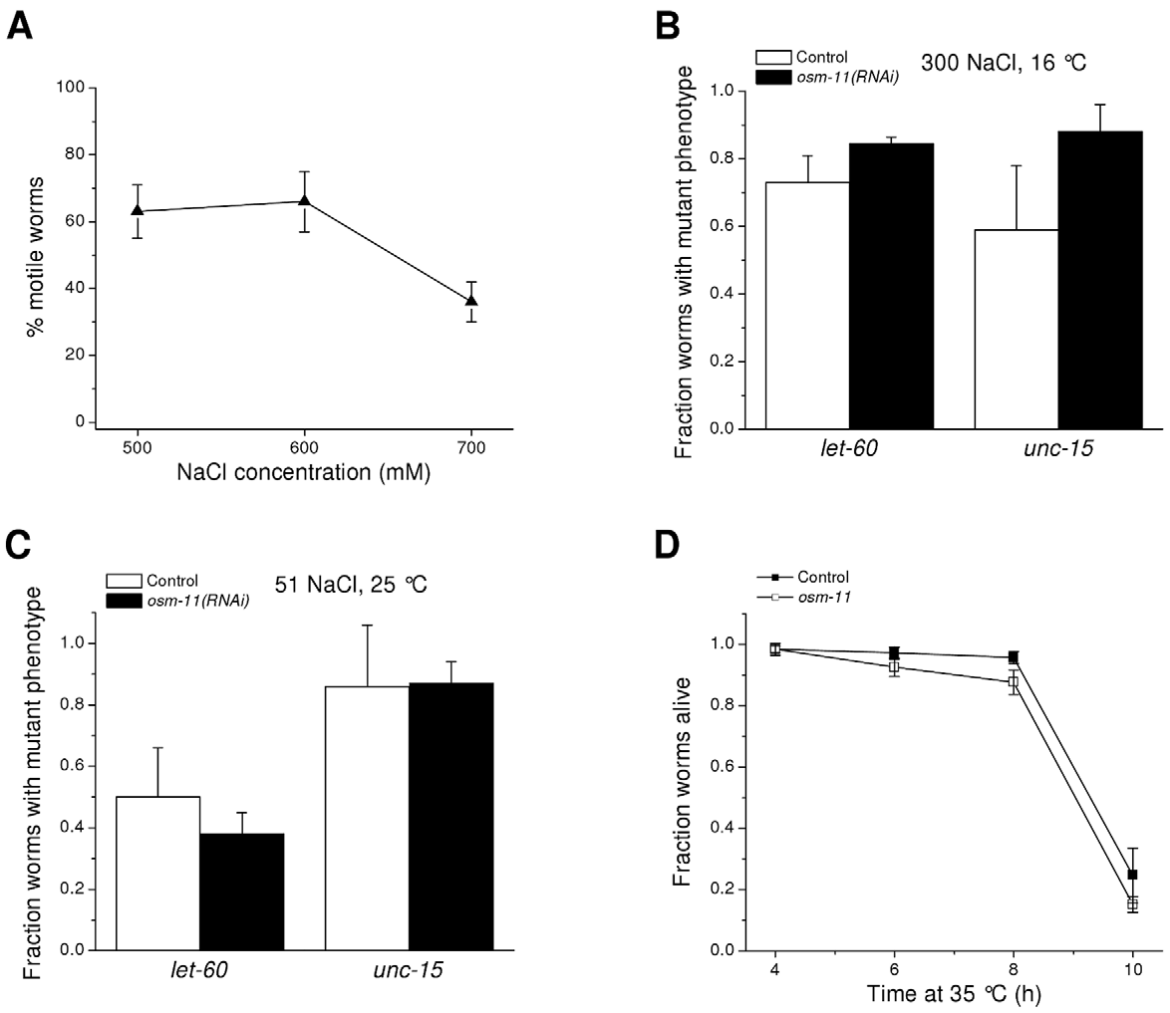
Effect of glycerol accumulation on protein misfolding. *A:* Effect of *osm-11* RNAi on motility of worms exposed acutely to 500, 600 or 700 mM NaCl. Motility of *osm-11(RNAi)* worms was not significantly (P>0.1) different from that of *osm-11* mutants exposed to the same NaCl levels (Figure 4A) confirming that silencing of the gene by RNAi elevates whole animal glycerol levels. *B, C:* Expression of mutant phenotypes in *let-60* and *unc-15* temperature sensitive mutant worms exposed to 300 mM NaCl at 16°C (*B*) or 51 mM NaCl at 25°C (*C*). *let-60* and *unc-15* encode a ras GTPase and paramyosin, respectively. As we have shown previously [6], hypertonic stress induces the mutant phenotype at low or permissive temperatures (i.e., 16°C) suggesting that water loss causes protein misfolding. Therefore, elevated glycerol levels do not suppress hypertonic stress- or temperature-induced expression of the *let-60* and *unc-15* mutant phenotypes. Control and *osm-11(RNAi)* worms were fed bacteria expressing a nonspecific dsRNA or *osm-11* dsRNA, respectively. (*n* = 6–9 experiments, each with 20–50 worms/experiments). *D:* Effect of RNAi knockdown of *osm-11* on survival during heat stress. Increased temperature causes protein misfolding and decreased survival [24], [25]. Elevated glycerol levels in *osm-11(RNAi)* worms do not enhance survival during heat shock. (*n* = 3–7 experiments with 15–40 worms/experiment).

We have shown previously that exposure to 300 mM NaCl induces mutant phenotypes in *let-60(ga89)* and *unc-15(el402)* ts worms maintained at the permissive temperature of 16°C [6]. These findings indicate that hypertonic stress induces protein misfolding in vivo. To assess the role of glycerol in preventing this misfolding, we fed *let-60(ga89)* and *unc-15(el402)* worms bacteria producing double-stranded RNA (dsRNA) homologous to *osm-11*. Compared to control animals fed a nonspecific dsRNA, *osm-11(RNAi)* worms maintained high levels of motility on growth plates containing 500, 600 and 700 mM NaCl (Figure 5A). Motility was not significantly (P>0.1) different from that of the *osm-11* mutants (see Figure 4A) indicating that glycerol levels, as expected, are elevated in *osm-11(RNAi)* worms.

*let-60(ga89)* and *unc-15(el402)* worms failed to lay eggs at NaCl concentrations of 400 mM and above. We therefore assessed the presence of the mutant phenotypes (i.e., egg hatching defects and larval arrest) in worms exposed to 300 mM NaCl at 16°C. As shown in Figure 5B, the fraction of mutant *let-60(ga89)*; *osm-11(RNAi)* and *unc-15(el402)*; *osm-11(RNAi)* worms was not significantly (P>0.7) different compared to worms fed nonspecific dsRNA. These results demonstrate that elevated glycerol levels do not prevent expression of the hypertonic stress-induced mutant phenotypes in *let-60* and *unc*-15 worms and suggests that glycerol is not capable of suppressing the misfolding of LET-60 and UNC-15 proteins in vivo.

Organic osmolytes protect some proteins from heat-induced misfolding in vitro and can suppress the thermosensitive phenotype of *dnaK* deletion in *E. coli* [22], [23]. We therefore examined the effect of elevated glycerol levels on the *let-60(ga89)* and *unc-15(el402)* mutant phenotypes induced by growing worms at the restrictive temperature of 25°C. RNAi silencing of *osm-11* had no significant (P>0.5) effect on induction of the mutant phenotypes by elevated growth temperature (Figure 5C).

Heat shock is well known to cause protein misfolding and activation of transcriptional pathways that increase molecular chaperone expression and resistance to heat stress [24], [25]. If glycerol functions as a chemical chaperone and plays an important role in preventing/reversing protein misfolding in vivo, *osm-11* mutant worms should survive heat shock better than wild type worms. To test this idea, we maintained worms at 35°C and monitored survival for 10 h. As shown in Figure 5D, there was no difference (P>0.1) in survival of *osm-11* mutants compared to wild type animals at any time point after induction of heat stress. Data shown in Figures 5B–D suggest that high levels of glycerol have little effect on temperature-induced protein misfolding.

In a recent genome-wide RNAi screen we identified 40 genes, termed Hos (HypertOnic Sensitive) genes, which are required for survival of *C. elegans* in hypertonic environments. Twenty Hos genes function in the destruction of damaged proteins [7]. It is conceivable that the suppression of protein damage observed in animals acclimated to mild hypertonic stress (Figures 3A and 4B–C) [6], [7] is due to increased protein degradation activity. We carried out four experiments to test this possibility. Chloroquine and MG-132 are potent inhibitors of lysosome and proteasome activity, respectively, in *C. elegans* [7], [26]. We have shown previously that Q35::YFP aggregation is increased in unstressed worms by treating them with these two drugs [7]. This demonstrates that protein degradation plays a role in suppressing spontaneous aggregate formation.

**Figure 6:**
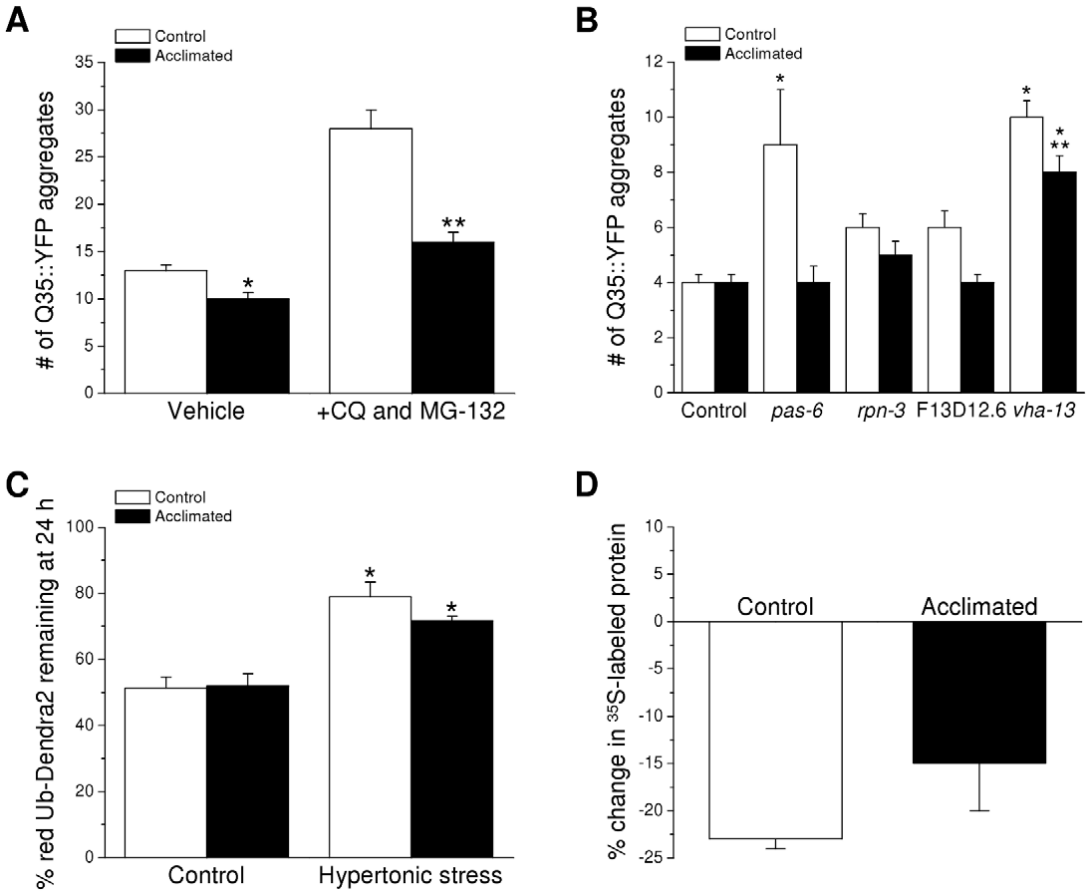
Effect of acclimation to mild hypertonic stress on protein degradation activity. *A:* Effect of treatment of control and acclimated worms with vehicle only (1% DMSO) or 20 mM chloroquine (CQ) and 100 µM MG-132 on spontaneous aggregation of Q35::YFP. (*n* = 9–15). *P<0.003 compared to vehicle-treated control worms. **P<0.0001 compared to drug-treated control worms. *B:* Effect of RNAi silencing of Hos genes on spontaneous Q35::YFP aggregation in control and acclimated worms. Animals were fed bacteria expressing nonspecific (control) dsRNA or dsRNA targeting proteasome (*pas-6* and *rpn-3*) and lysosome (*vha-13*) components, or a putative lysosomal serine carboxypeptidase (F13D12.6). (n = 16–51). *P<0.001 compared to control or acclimated worms fed a nonspecific dsRNA. **P<0.03 compared to unacclimated *vha-13(RNAi)* worms. *C:* Percent of red mutant ubiquitin (UbG76V) tagged Dendra2 remaining in body wall muscle cells 24 h after photoconversion in control and 200 mM NaCl acclimated worms exposed to control or hypertonic growth media. Control and acclimated animals were exposed to 400 mM and 600 mM NaCl, respectively. (*n* = 3–8). *P<0.01 compared to unstressed worms. *D:* Percent change in ^35^S-methionine labeled total protein levels in control and acclimated worms treated with 500 µg/ml of cycloheximide for 6 h to inhibit protein synthesis. (*n* = 3).

To assess the role of role of lysosome and proteasome activity on Q35::YFP aggregation in control and acclimated worms, synchronized L1 larvae were grown to adulthood for 2 days on control (51 mM NaCl) or 200 mM NaCl agar plates. They were then transferred to 51 or 200 mM NaCl plates containing vehicle only (1% DMSO) or 20 mM chloroquine and 100 µM MG-132 and Q35::YFP aggregates were quantified 48 h after transfer. As shown in Figure 6A, treatment of control worms with chloroquine and MG-132 increased the mean number of Q35::YFP aggregates nearly 2.2-fold (P<0.0001) from 13 to 28. In acclimated worms treated with vehicle only the mean number of Q35::YFP aggregates was 10 compared to 13 observed in vehicle-treated control animals. While the difference was statistically significant (P<0.003), it was not reproducible (compare to Figures 1B and 6B) and is within the range of variability typically seen in these measurements.

Treatment of acclimated worms with chloroquine and MG-132 increased the mean number of aggregates to 16 (P<0.0001), which was ∼40% lower (P<0.0001) than that observed in drug-treated controls. As we have suggested previously [7], these results indicate that protein degradation via lysosomes and proteasomes plays a role in suppressing spontaneous Q35::YFP aggregation. However, the striking reduction of spontaneous aggregation in acclimated worms treated with lysosome and proteasome inhibitors suggests that acclimation to mild hypertonic stress suppresses protein aggregation by mechanisms that are independent from protein degradation.

We also inhibited lysosome and proteasome activity using RNAi knockdown of Hos genes that encode proteasome or lysosome components and quantified the effect on Q35::YFP aggregation. The Hos genes tested were *pas-6* and *rpn-3*, which encode components of the 26S proteasome, *vha-13*, which encodes a subunit of the vacuolar proton-translocating ATPase, and F13D12.6, which encodes a putative lysosomal serine carboxypeptidase. RNAi was performed by transferring synchronized L1 larvae to control (51 mM NaCl) or acclimation (200 mM NaCl) agar plates seeded with bacteria producing dsRNA targeting one of the Hos genes. Aggregates were quantified 72 h after transfer.

As shown in Figure 6B, RNAi of *pas-6* and *vha-13* caused significant (P<0.001) increases in Q35::YFP aggregation in unacclimated worms relative to control animals fed bacteria expressing a nonspecific dsRNA. In contrast, only knockdown of *vha-13* increased (P<0.001) aggregation in worms acclimated to 200 mM NaCl. When compared to control worms, the number of aggregates observed in acclimated *vha-13(RNAi)* animals was significantly (P<0.03) lower. These data are consistent with chloroquine/MG-132 studies (Figure 6A) and suggest that suppression of protein damage in acclimated worms is not mediated by increased activity of protein degradation pathway.

Interpretation of the results shown in Figure 6A–B assumes near complete inhibition of proteasome and lysosome activity by chloroquine and MG-132 and by RNAi. To more directly assess whether protein degradation capacity is increased in acclimated worms, we quantified proteasome activity using a worm strain expressing in their body wall muscle cells the green fluorescent protein Dendra2 tagged with the mutant ubiquitin UbG76V. UbG76V is not cleaved off of proteins by ubiquitin hydrolases, resulting in polyubiquitination of UbG76V-Dendra2 and subsequent targeting of the protein to proteasomes for degradation. Excitation of Dendra2 with 405 nm light converts its green fluorescence to red, thus generating a pool of mature, red fluorescent Dendra2 distinct from any newly translated, green fluorescent Dendra2. Decreases in red fluorescence therefore reflect proteasome activity [27].

Figure 6C shows the amount of red UbG76V-Dendra2 remaining in body wall muscle cells 24 h after photoconversion under control conditions and during exposure to hypertonic stress. To induce similar degrees of water loss, control worms were exposed to 400 mM NaCl while acclimated animals were exposed to 600 mM NaCl (Figure 4A). There were no significant differences (P>0.3) between red Dendra2 levels in control and acclimated animals under either control or hypertonic stress conditions demonstrating that proteasome activity is not upregulated in acclimated worms. Interestingly, however, hypertonic stress caused a significant (P<0.01) inhibition of red Dendra2 degradation. A likely explanation for these results is that hypertonicity-induced protein damage increases the load on the proteasomal degradation system resulting in a slowing of Dendra2 removal.

To quantify total protein degradation capacity, cellular proteins in control worms and worms acclimated to 200 mM NaCl were labeled with ^35^S-methionine. Immediately after the intestinal purge step of the radiolabeling procedure, worms were either frozen for protein extraction or transferred for 6 h to control or 200 mM NaCl plates containing 500 µg/ml cycloheximide. This concentration of cycloheximide has been shown by Kourtis and Tavernarakis [28] to effectively block protein synthesis in *C. elegans*. As shown in Figure 6D, ^35^S-labeled total protein levels in control and acclimated worms dropped by 23% and 15%, respectively, after inhibition of protein synthesis by cycloheximide. These reductions in labeled protein levels were not significantly (P>0.2) different demonstrating that rates of protein degradation are similar in control and acclimated worms.

Data in Figures 1, 2, 3, 4, 5, 6 demonstrate that neither glycerol accumulation nor upregulation of lysosome/proteasome activity are responsible for decreases in hypertonicity-induced protein damage observed in worms acclimated to mild hypertonic stress. Therefore, acclimation most likely increases molecular chaperoning capacity. Increased chaperone capacity can be brought about by increases in the activity of molecular chaperones and/or by decreases in protein synthesis.

Reductions in protein synthesis decrease the number of nascent and newly synthesized proteins that are subjected to misfolding and aggregation. This reduction in turn is expected to decrease the load on the molecular chaperone and protein degradation network thereby increasing their availability for minimizing, reversing and/or removing damage to existing proteins. Inhibition of protein synthesis will also free up energy resources for use in other cellular processes including protein repair and degradation.

**Figure 7:**
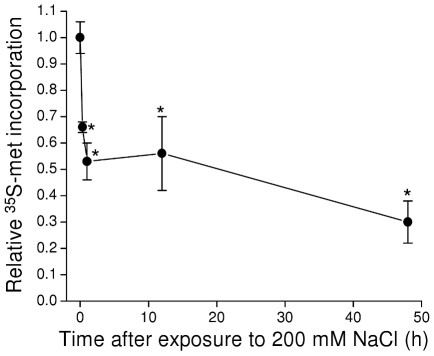
Effect of acute hypertonic stress on ^35^S-methionine incorporation into total protein. Worms were transferred to 200 mM NaCl agar plates at time 0 and ^35^S-methionine incorporation into total protein was quantified 20 min and 1, 12 and 48 h after transfer. Values are expressed relative to unstressed control worms (i.e., time 0). (*n* = 3). *P<0.05 compared to control worms.

Numerous environmental and physiological stressors inhibit protein synthesis in diverse cell types and organisms [29], [30]. We quantified ^35^S-methionine incorporation into total protein to assess the effects of hypertonic stress on protein translation in *C. elegans*. As shown in Figure 7, a striking drop in ^35^S-methionine incorporation was detected with as little as 20 min of exposure to 200 mM NaCl. Maximal inhibition was observed within 1 h, and translation remained inhibited at this level for at least 48 h.

While unlikely, it is possible that the reduced incorporation of ^35^S-methionine into total protein observed in hypertonically stressed worms was due to an increased rate of protein degradation with subsequent loss of the labeled methionine. Total protein degradation is similar in control and fully acclimated worms (Figure 6D). To determine whether protein degradation changes during the acclimation process, we measured the decline in ^35^S-methionine labeled protein levels in worms treated with 500 µg/ml cycloheximide. Protein degradation was examined at a time point when systemic volume recovery was complete [11], [12] to avoid the more extreme stress of severe water loss combined with cycloheximide treatment. Worms grown on 51 mM NaCl were labeled with ^35^S-methionine and then exposed to either 51 or 200 mM NaCl. After 15 h, the worms were frozen or transferred to 51 or 200 mM NaCl agar plates containing cycloheximide for 6 h. As expected, ^35^S-methionine labeled protein levels declined in cycloheximide-treated worms. The mean ± S.E. percent change observed was −36±4% and −40±9% (n = 4) for worms exposed to 51 or 200 mM NaCl, respectively. These values were not significantly (P>0.6) different demonstrating that rates of protein degradation are similar in control worms and worms exposed acutely to 200 mM NaCl. Thus, the reduced ^35^S-methionine incorporation into total protein observed in worms acclimating to hypertonic stress reflects a reduced rate of protein synthesis rather than increased degradation.

**Figure 8:**
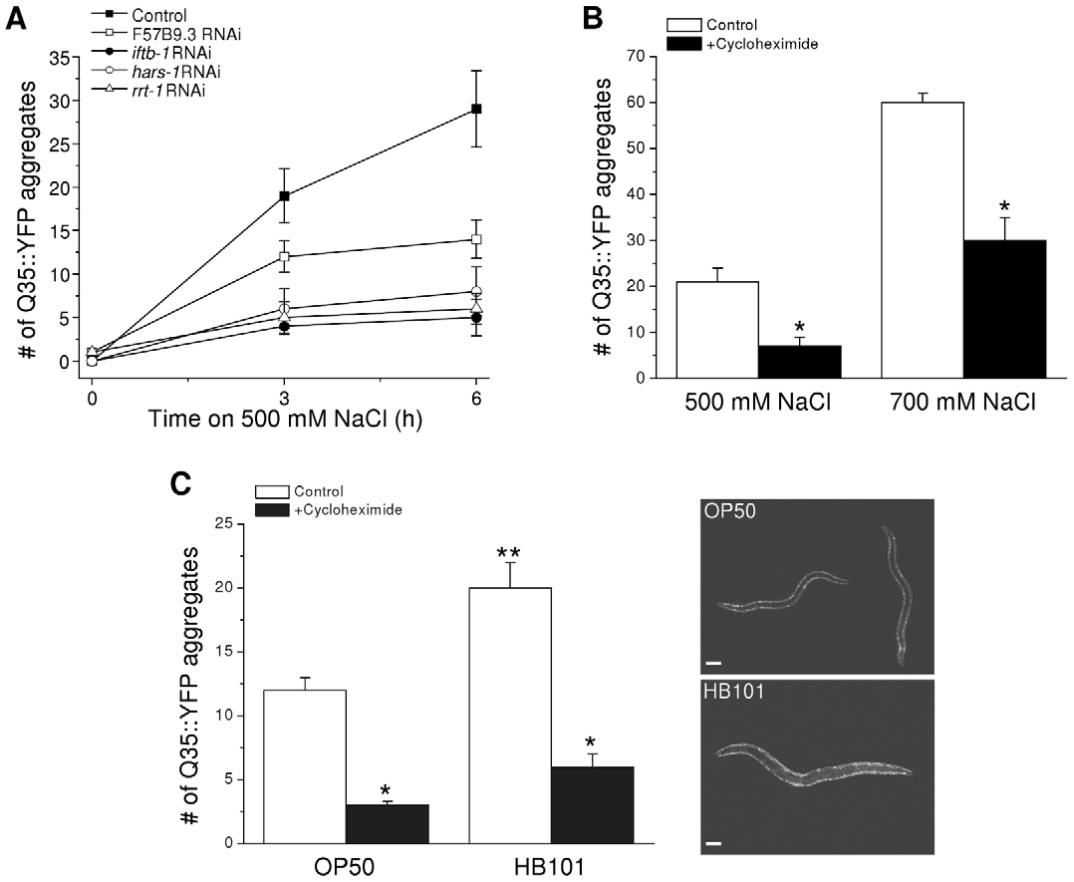
Effect of inhibition of protein synthesis on hypertonic stress-induced Q35::YFP aggregation. *A:* Hypertonic stress-induced Q35::YFP aggregates in worms fed bacteria producing a nonspecific dsRNA (control) or bacteria producing dsRNA targeting eukaryotic translation initiation factors (F57B9.3 and *iftb-1*) or amino-acyl tRNA synthetases (*hars-1* and *rrt-1*). The number of Q35::YFP aggregates was significantly (P<0.002) lower at both 3 and 6 h in worms fed dsRNA targeting *iftb-1*, *hars-1* and *rrt-1*. Aggregates were reduced significantly *(P<0.004) at 6 h in worms fed RNAi targeting F57B9.3. (n = 15). *B:* Hypertonic stress-induced Q35::YFP aggregates in control worms and worms exposed to 500 µg/ml cycloheximide. Worms were exposed to cycloheximide in control growth medium for 15 min and then transferred to 500 or 700 mM agar containing cycloheximide. After 1 h worms were returned to control medium without the drug and aggregates quantified 2.5–3 h later. (n = 8–12). *P<0.0005 compared to untreated control worms. *C:* Left panel, hypertonic stress-induced Q35::YFP aggregation in worms fed OP50 or HB101 bacteria. Worms were exposed to 400 mM NaCl for 1 h. The protocol for cycloheximide treatment was the same as that described in *B*. (n = 14–27). *P<0.0001 compared to control worms without cycloheximide treatment. **P<0.0009 compared to OP50 fed worms. Right panel, representative fluorescent micrographs of OP50 and HB101 fed worms exposed to 400 mM NaCl for 1 h. Scale bar is 100 µm.

To test whether inhibition of protein synthesis reduces hypertonic stress-induced protein damage we silenced the expression of genes that play essential roles in translation. Worms were fed bacteria expressing dsRNA homologous to genes encoding arginyl and histidyl amino-acyl tRNA synthetases (*rrt-1* and *hars-1*, respectively), and the eukaryotic translation initiation factors eIF2β (*iftb-1*) and eIF4A (F57B9.3). Knockdown of these genes inhibits ^35^S-methionine incorporation into total protein by 50–80% ([31], [32] and Lee and Strange, unpublished observations). As shown in Figure 8A, RNAi of *rrt-1*, *hars-1*, and *iftb-1* inhibited hypertonicity-induced Q35::YFP aggregation by ∼70–80% 3 and 6 h after exposure to 500 mM NaCl (P<0.002). RNAi of F57B9.3 inhibited aggregation significantly (P<0.004) by ∼50% at 6 h only.

Loss of *rrt-1*, *hars-1*, *iftb-1* or F57B9.3 also activates *gpdh-1* expression ([12] and Lee and Strange, unpublished observations). Because constitutive activation of *gpdh-1* usually results in elevation of whole animal glycerol levels ([12] and Figure 1A), it is conceivable that the inhibition of Q35::YFP aggregation shown in Figure 8B is due to reduced water loss and shrinkage in worms exposed to 500 mM NaCl. To test this possibility, we carried out motility assays. 15±3%, 20±6% and 20±11% (n = 4–7 experiments) of worms fed bacteria expressing nonspecific, *hars-1* or *rrt-1* dsRNA remained motile for up to 1 h when placed on 500 mM NaCl. These values were not significantly (P>0.5) different. *iftb-1(RNAi)* and *F57B9.3(RNAi)* showed significantly (P<0.008) reduced motility under these conditions (mean ± S.E. motility was 0±0% and 2±2% in *iftb-1(RNAi)* and *F57B9.3(RNAi)* worms, respectively; n = 5 experiments). The reason for the reduced motility is unclear. However, the results demonstrate that the reduction of Q35::YFP aggregation in *rrt-1*, *hars-1*, *iftb-1* and F57B9.3 RNAi worms is due to inhibition of protein synthesis per se rather than reduced water loss and shrinkage.

It is conceivable that chronic inhibition of protein synthesis by RNAi reduces Q35::YFP aggregation simply by reducing the cellular concentration of Q35::YFP protein and/or by increasing the expression of components of the chaperoning/degradation network that may minimize aggregate formation. To address these possibilities, we reduced translation acutely by treating Q35::YFP worms with 500 µg/ml of cycloheximide for 15 min followed by a 1 h exposure to 500 or 700 mM NaCl with cycloheximide present. Worms were then transferred back to control growth medium without drug and Q35::YFP aggregates were quantified 2.5–3 h later. As shown in Figure 8B, cycloheximide treatment reduced the number of Q35::YFP aggregates ∼66% and ∼50% (P<0.0005) in worms exposed to 500 mM and 700 mM NaCl, respectively.

Acute inhibition of protein synthesis could conceivably reduce cellular protein levels and hence macromolecular crowding, which in turn could reduce Q35::YFP aggregation. To test this possibility, we measured total protein in worms exposed to 400 mM NaCl in the presence and absence of cycloheximide. The mean ± S.E. protein levels in control and cycloheximide-treated worms were 0.66±0.04 µg/worm (n = 5) and 0.48±0.15 µg/worm (n = 3), respectively, and were not significantly (P>0.4) different.

The cycloheximide-induced reduction in Q35::YFP aggregation shown in Figure 8B could be due to increases in the availability of molecular chaperone and/or protein degradation networks for suppression of hypertonic stress-induced protein damage. To test whether protein degradation plays a role in suppressing Q35::YFP aggregation, we pretreated worms for 3 h with 20 mM chloroquine and 100 µM MG-132. Worms were then exposed continuously to these drugs during the 15 min cycloheximide pretreatment, during exposure to 500 mM NaCl with cycloheximide, and during the 2.5–3 h recovery period. In worms treated with cycloheximide alone or cycloheximide, chloroquine and MG-132, we observed 6±2 and 8±2 (mean ± S.E.; n = 10 worms) hypertonic stress-induced Q35::YFP aggregates, respectively. There was no significant (P>0.5) difference in the number of aggregates observed in the two groups of animals. These results suggest that the suppression of Q35::YFP aggregation induced by cycloheximide is due to the activity of molecular chaperones rather than protein degradation mechanisms.

If inhibition of protein synthesis reduces Q35::YFP aggregation during hypertonic stress, the converse is expected to be true: increased protein production and cellular protein levels should increase aggregate formation. To the best of our knowledge, no experimental maneuver has been identified that acutely stimulates protein synthesis in *C. elegans*. However, recent studies by So et al. [33] have shown that feeding *C. elegans* the *E. coli* strain HB101 increases body size 1.6-fold. This increase is due entirely to cell enlargement with a concomitant increase in worm protein content of ∼60%. Numerous studies in diverse organisms and cell types have shown that increases in cell size and protein content are tightly coupled to increases in protein synthesis (e.g., [34]–[37]).

To assess the effects of intracellular protein levels on Q35::YFP aggregation, we fed worms either OP50 or HB101 *E. coli* strains. Assuming that worm shape approximates a cylinder, we estimated that HB101 fed worms were ∼1.7-fold larger than OP50 fed animals (data not shown), which is similar to that observed by So et al. [33]. Interestingly, when exposed to NaCl concentrations of 500 mM or greater, HB101 fed worms survived poorly compared to OP50 fed animals (data not shown). However, survival was similar when worms were exposed to 400 mM NaCl for 24 h (90±7% and 91±1% in HB101 and OP50 fed worms, respectively; n = 15–20 worms and 3 independent experiments).

OP50 and HB101 fed worms were exposed to 400 mM NaCl for 1 h and then allowed to recover on 51 mM for 2–2.5 h before Q35::YFP aggregates were quantified. As shown in Figure 8C, the number of aggregates was 1.7-fold higher (P<0.0009) in HB101 fed worms. The results were even more striking when worms were exposed continuously to 400 mM NaCl for 24 h. Worms fed OP50 bacteria had 13±2 aggregates (n = 9) while in HB101 fed worms the number of aggregates was 2.8-fold higher (37±3 aggregates; n = 9; P<0.0001).

The higher levels of Q35::YFP aggregation in HB101 fed worms could be due to increased Q35::YFP concentration. To assess this, we quantified Q35::YFP fluorescence intensity in body wall muscle prior to inducing aggregation by exposing worms to hypertonic stress. Fluorescence was measured in a 50×98 pixel rectangular region of interest in six different areas of single worms. The box was placed so that the body wall extended from an upper corner to the opposite lower corner. This approach ensured that similar amounts of body wall were present in each measurement box. Mean ± S.E. pixel intensity was 4107±140 and 3905±183 (n = 8 worms) in OP50 and HB101 fed worms, respectively. These values were not significantly (P>0.4) different indicating that increased Q35::YFP aggregation is not due simply to increased concentration of the aggregating protein.

Finally, we assessed the effect of acute inhibition of protein synthesis on Q35::YFP aggregation in worms fed HB101 bacteria. Worms were pre-treated with 500 µg/ml cycloheximide for 15 min, exposed to 400 mM NaCl for 1 h in the presence of the drug and then allowed to recover for 2–2.5 h on control medium before Q35::YFP aggregates were quantified. Cycloheximide treatment reduced Q35::YFP aggregate formation 75% and 70% (P<0.0001) in OP50 and HB101 fed worms, respectively (Figure 8C).

Consistent with previous findings [33], we found that protein levels in HB101 bacteria fed worms (1.04±0.2 µg/worm, n = 4) were significantly (P<0.04) higher than in worms fed OP50 bacteria (0.66±0.02 µg/worm, n = 6). Acute cycloheximide treatment had no significant (P>0.4) effect on protein levels in either group of animals (OP50 bacteria fed worms = 0.48±0.15 µg/worm, n = 3; HB101 bacteria fed worms = 0.89±0.11 µg/worm, n = 5). Taken together, data in Figure 8 demonstrate that increased protein expression results in increased hypertonic stress-induced protein damage.

## Discussion

In vitro studies have led to the widely held assumption that hypertonic stress damages animal cell proteins in vivo (e.g., [3]). However, with the exception of our recent work in *C. elegans*, there are relatively few studies that have addressed this problem directly. We have shown that hypertonic stress causes rapid and widespread damage to diverse proteins in worms [6], [7], and that the ability of *C. elegans* to survive in hypertonic environments depends critically on the activity of genes that function to destroy damaged proteins [7].

Work from numerous labs in diverse animals and cell culture models has shown that acclimation to low levels of environmental stressors such as heat, heavy metals or hypertonicity increases survival during exposure to more extreme levels of the same stress (e.g., [38]–[40]). Similarly, acclimation of *C. elegans* to relatively mild hypertonic stress increases survival under more extreme hypertonic conditions [11]. Importantly, acclimation also activates mechanisms that function to suppress hypertonicity-induced protein damage [6], [7].

Understanding the mechanisms that protect proteins from environmental stressors and that repair and degrade damaged proteins during cellular stress has important implications for understanding and treating numerous diseases and the pathophysiology associated with aging [1], [41]. *C. elegans* provides multiple advantages for defining these mechanisms including experimental speed and economy, and genetic and molecular tractability. The *C. elegans* hypertonic stress response is a particularly powerful model for defining stress-associated protein quality control mechanisms due to the extent and speed at which water loss causes damage to diverse proteins and the relative ease of quantifying these changes in diverse genetic backgrounds and under various experimental conditions [6], [7].

As discussed earlier, there are three physiological processes that could play a role in suppressing hypertonicity-induced protein damage in acclimated worms: 1) accumulation of the organic osmolyte glycerol, 2) increased protein degradation activity, and/or 3) increased capacity of the molecular chaperone network. It was surprising and interesting to find that glycerol, the major organic osmolyte accumulated by *C. elegans* in response to water loss [11], [12], has no obvious impact on protein aggregation (Figures 1A, 3A, 4B–C), Q35::YFP toxicity (Figure 2C) or misfolding of at least two proteins (Figure 5). Organic osmolytes are widely viewed to function as chemical chaperones that aid in stabilizing native protein conformation and in assisting the refolding of denatured proteins [20], [21]. The vast majority of work that supports this idea comes from in vitro studies. A few in vivo studies in bacteria have characterized the role of organic osmolytes in inhibiting spontaneous aggregation of transgenic proteins. For example, Ignatova and Gierasch [42] demonstrated that proline inhibits aggregation of the aggregation-prone P39A cellular retinoic acid binding protein in *E. coli*, and Schultz et al. [43] have proposed that trehalose inhibits aggregation of several *E. coli* expressed recombinant proteins. However, to the best of our knowledge, there are no previous in vivo studies that have directly assessed the role of organic osmolytes in protecting proteins from hypertonic stress-induced damage.

Both in vitro and in vivo studies have also shown that organic osmolytes protect proteins from heat-induced damage (e.g., [22], [23]). However, high glycerol levels do not suppress elevated temperature-induced expression of the ts phenotype in *let-60* and *unc-15* mutants (Figure 5C) and do not confer increased resistance to heat shock (Figure 5D). Taken together, our studies raise questions about the physiological roles of glycerol and possibly other organic osmolytes in protecting proteins from stress-induced damage.

Interestingly, the presence of high glycerol levels in *osm-11* mutants is associated with increased hypertonic stress-induced aggregation of Q35::YFP (Figure 3A) even though these animals lose less water than control worms under comparable conditions (Figures 3B, 4A). It is important to note that despite the widely held view that organic osmolytes stabilize native protein structure, these solutes can have no protective effect or can even enhance protein misfolding and aggregation in vitro. For example, betaine concentrations of 5–20 mM cause GST-GFP to misfold and aggregate [44]. Relatively low concentrations (100 mM) of proline increase heat-induced aggregation of glycogen phosphorylase *b* [45]. Molar concentrations of glycerol have little or no effect on folding of RNase T1 [46], and do not reverse inhibition or prevent aggregation of unfolded lysozyme [47]. The presence of glycerol in solution increases thermal inactivation of phosphofructokinase [48]. These findings together with our results indicate that extensive in vivo studies of organic osmolyte function are required to define the precise role of these solutes during osmotic stress.

Our recent genome-wide RNAi screen identified 40 genes that are required for survival of *C. elegans* in hypertonic environments. Twenty of these genes encode proteins that detect, transport, and degrade damaged proteins, including components of the ubiquitin-proteasome system, endosomal sorting complexes, and lysosomes [7]. Upregulation of cellular protein degradation capacity, however, is not responsible for the suppression of protein damage observed in acclimated worms (Figure 6).

Our results suggest strongly that the primary mechanism limiting protein damage in acclimated worms exposed to more extreme hypertonic stress is increased molecular chaperone capacity. This increased capacity could be brought about in two ways. First, as shown for many stressors including osmotic stress [9], the expression of molecular chaperones may be increased. However, microarray studies carried out by Rohlfing et al. [49] demonstrated that exposure of *C. elegans* to cadmium increases the expression of 14 heat shock proteins (HSP) whereas no HSPs are induced by osmotic stress and some show reduced transcription. It is important to note though that increased expression of chaperones could be brought about by enhanced translation of chaperone mRNA [50] and/or chaperone activity may be increased by post-translational modifications such as phosphorylation (e.g., [51], [52]). Additional studies are required to assess the role of these processes in the *C. elegans* osmotic stress response.

Molecular chaperone capacity can also be increased by decreasing the load on the molecular chaperone network. Acclimation of *C. elegans* to mild hypertonic stress inhibits protein synthesis ∼50–70% (Figure 7). Inhibiting translation reduces energy consumption, the total number of cellular proteins that may be damaged by the stressor, and the number of nascent proteins prone to aggregation and that require co-translational chaperone-mediated folding. This in turn is expected to free up energy resources, molecular chaperones and protein degradation machinery that could be used to minimize and reverse damage to existing proteins or remove damaged proteins from the cell.

While the idea that inhibition of translation makes cellular resources available that could be used to minimize protein damage is an attractive and widely cited one [29], [30], there is little direct evidence to support it. For example, chronic inhibition of protein synthesis extends lifespan in numerous model systems [53]. Two studies in *C. elegans* have shown that inhibition of translation also increases thermal [32] and oxidative [54] stress resistance. Increased stress resistance may be due to the increased ability of cells to reduce protein damage. King et al. [55] have shown that inhibition of protein synthesis for 20 h by rapamycin or cycloheximide inhibits spontaneous polyglutamine aggregation in mammalian cells. However, they conclude that this is due to a reduction in the concentration of mutant protein required for nucleation and subsequent aggregation to occur.

Other studies suggest that reductions in protein synthesis have no effect on protein damage or actually increase it. Moulder et al. [56] showed that cycloheximide does not alter spontaneous aggregation-induced polyglutamine-GFP toxicity in cultured mammalian neurons. In *C. elegans*, silencing of genes required for protein synthesis, including genes encoding translation initiation factors, induces early onset of spontaneous polyglutamine-YFP aggregation [57].

Our studies demonstrate that both chronic and acute inhibition of protein synthesis dramatically inhibit hypertonic stress-induced aggregation of Q35::YFP (Figure 8). To the best of our knowledge, this is the first direct demonstration that the rate of protein synthesis reduces protein damage brought about by an environmental stressor. The inability of inhibitors of proteasome and lysosome activity to reverse the protective effect of cycloheximide (see Results) suggests that molecular chaperone activity is primarily responsible for suppressing hypertonic stress-induced Q35::YFP aggregation during acute inhibition of protein synthesis.

Increased transcriptional expression of *gpdh-1* is required for glycerol accumulation when worms are exposed to hypertonic environments [12]. Genome-wide RNAi screening identified 122 *rgpd* (regulators of *gpdh-1*) genes that when silenced induce constitutive expression of *gpdh-1* and glycerol accumulation. *rgpd* gene functions fall into several well defined categories. The largest *rgpd* gene class (45% or 55/122 genes) comprises genes that carry out highly conserved and essential roles in protein synthesis including aminoacyl-tRNA synthetases and eukaryotic translation initiation factors (eIFs) [12]. Silencing of many of these conserved protein synthesis genes is known (e.g., [31], [32] and Lee and Strange, unpublished observations) or is expected to inhibit protein translation. Hypertonicity-induced inhibition of protein synthesis therefore not only reduces protein damage, but also appears to function as a signal that activates stress response pathways required for survival of worms in hypertonic conditions. *C. elegans* thus provides a unique model for developing an integrated systems level understanding of how stressors control protein translation and coordinate that control with maintenance of proteostasis and activation of selective stress protective mechanisms.

It is interesting to compare our results with those of Nollen et al. [57]. These investigators carried out a genome-wide RNAi screen to identify suppressors of spontaneous Q35::YFP aggregation. They observed that aging-induced aggregation is enhanced by RNAi knockdown of eukaryotic translation initiation factors including *iftb-1* and F57B9.3. In contrast, *iftb-1* or F57B9.3 knockdown reduces hypertonic stress-induced Q35::YFP aggregate formation (Figure 8A). These observations as well our data showing that acclimation to mild hypertonic stress suppresses hypertonicity-induced but has no reproducible effect on spontaneous Q35::YFP aggregation (see Figures 1B and 3A) suggest that the two aggregation processes are distinct. Our findings underscore the importance of defining the mechanisms that cells use under different physiological and pathophysiological conditions to prevent and reverse protein damage, and in determining whether these different mechanisms could be therapeutically manipulated to slow or reverse protein damage associated with disease and aging.

## Materials and Methods

### *C. elegans* strains

The following strains were used: N2 Bristol (wild type), AM140 rmIs132[*Punc-54*::Q35::YFP], MT3643 *osm-11(n1604)*, VP586 *osm-11(n1604)*; rmIs132[*Punc-54*::Q35::YFP], VP332 *gpdh-1(kb24); gpdh-2(kb33)*, CB1402 *unc-15(e1402)*, SD551 *let-60(ga89)*, and YD3 *xzEx3*[*Punc-54::UbG76V::Dendra2*]. Unless noted otherwise, worms were cultured at 20°C using standard methods. For acclimation studies, eggs were placed onto 200 mM NaCl agar plates without food overnight for synchronization to L1 larvae. L1 larvae were then transferred to 200 mM NaCl plates seeded with bacteria.

### Measurement of whole worm glycerol levels

Worms were washed in liquid nematode growth medium (NGM) buffer to remove bacteria and frozen at −80°C. Glycerol was measured as described previously, with some modifications [11]. Frozen worms were sonicated in liquid NGM media on ice. Insoluble debris was removed from these lysates by centrifugation, and a portion of the supernatant was assayed for total protein concentration using a bicinchoninic acid (BCA) assay (Pierce Biotechnology, Rockford, IL). 1 N perchloric acid (PCA) was added to the remaining lysate to precipitate proteins, and the proteins were removed by centrifugation. The supernatant, which contained glycerol, was titrated to a pH of 6.7–7.5 with 5 N KOH in 61.5 mM K_2_HPO_4_ and 38.5 mM KH_2_PO_4_. Glycerol was measured in the neutralized supernatants with a commercial kit according to manufacturer's protocols (R-Biopharm, Marshall, MI). Glycerol levels were expressed relative to total protein content.

### Quantification of Q35::YFP aggregates

Q35::YFP aggregates were counted manually from digital images as described previously [15]. Images of transgenic worms were obtained with a Zeiss Stemi SV11 microscope fitted with a CCD-100 DAGE-MTI camera set to excite and view GFP. During imaging, worms were immobilized by chilling agar plates on ice.

Aging-induced Q35::YFP aggregation was quantified by transferring young adult stage worms to growth plates containing 50 µg/ml fluorodeoxyuridine to inhibit the development of offspring [58]. Hypertonicity-induced aggregates were quantified in worms transferred at the late L4/young adult stage to growth plates with elevated NaCl concentrations.

### Confocal microscopy and FRAP analysis

A Zeiss LSM510-Meta confocal microscope equipped with Plan-Neofluar 40×/1.3 NA and Plan-Apochromat 63×/1.4 NA oil objective lenses (Carl Zeiss MicroImaging, Thornwood, NY) was used for imaging. FRAP analysis was performed by photobleaching regions of body wall muscle cells with 50–150 iterations of an Argon laser set at 100% power and 514 nm for bleaching of YFP. Images were taken every 3 s for up to 60 s after photobleaching and fluorescence intensity was measured with ImageJ software (National Institutes of Health).

### Quantification of Q35::YFP aggregate toxicity

To assess the toxicity of Q35::YFP aggregates in body wall muscle cells, worm mobility was measured as described previously [15]. Briefly, single worms were placed on a fresh lawn of OP50 bacteria and then removed after 3 to 10 minutes. Brightfield images of the lawns were obtained with a Zeiss Stemi 2000 microscope fitted with a CCD-100 DAGE-MTI camera, or a Zeiss V12 M2Bio microscope fitted with a Zeiss AxioCam MRm cooled CCD camera. The length of tracks made by worms was measured with Image J software.

### Analysis of endogenous insoluble proteins

Isolation of insoluble proteins was carried out as described previously [6]. Briefly, worms were washed in NGM solution isotonic to the agar medium on which they were grown. Washed worms were transferred to a buffer containing NaCl at a concentration equivalent to the growth medium and 100 mM MES, 1 mM EGTA, 0.1 mM EDTA, 0.5 mM MgSO_4_, 20 mM NaF, drip frozen in liquid nitrogen, and ground to a powder with a mortar and pestle. Immediately upon thawing, 10 µl of the ground material was taken for analysis of total protein concentration using a bicinchoninic acid (BCA) protein assay (Pierce Biotechnology, Rockford, IL). Two additional 60 µl aliquots were placed in either a solubilization buffer (8 M urea, 2% SDS, 50 mM DTT, 50 mM Tris, Roche complete protease inhibitor, pH 7.4) for total protein determination, or RIPA buffer (50 mM Tris, 150 mM NaCl, 5 mM EDTA, 0.5% SDS, 0.5% SDO, 1% NP-40, Roche complete protease inhibitor, pH 8). Insoluble proteins were isolated from samples in RIPA buffer by centrifugation at 16,100 g for 10 min. After supernatant removal, the insoluble protein pellet was resuspended in 100 µl of RIPA buffer, centrifuged a second time and then solubilized in solubilization buffer.

Protein samples were analyzed by SDS-PAGE. Gels were stained with Bio-Safe Coomassie (Bio-Rad, Hercules, CA), and the amount of protein present in each lane was quantified with ImageJ software. To assess changes in the amount of insoluble protein, total protein lanes were loaded with a sample volume containing 20 µg of protein and the equivalent sample volume was used to load the corresponding insoluble protein lane.

### Photoconversion and quantification of Dendra2 fluorescence

Worms were anesthetized in isotonic NGM buffer containing 0.1% Tricaine and 0.01% tetramisole, mounted on 2% agarose pads on glass slides and imaged using a Zeiss LSM510-Meta confocal microscope with Plan-Neofluar 10×/0.3 objective. Photoconversion of Dendra2 was performed with 20 iterations of a 405 nm, 30 mW diode laser at 50% power. Green and red populations of Dendra2 were imaged using a 488 nm, 30 mW Argon laser at 1% power and a 543 nm, 1 mW HeNe laser at 10% power, respectively. Fluorescence intensity was measured with ImageJ software.

### Measurement of worm motility during acute osmotic stress

An internal hydrostatic pressure is required for motility in *C. elegans*. Water loss during hypertonic stress lowers hydrostatic pressure resulting in partial or complete paralysis until fluid balance is restored. To indirectly assess the degree of water loss under hypertonic conditions, we carried out motility assays similar to those described by Solomon et al. [59]. Briefly, worms were placed on high NaCl plates in the middle of a 7 mm circle. Motile worms were defined as those that moved to the outside of the circle within 30 min after transfer.

### ^35^S-methionine labeling of total cellular protein

Incorporation of ^35^S-methionine into total protein was used to assess rates of protein synthesis and degradation. Radiolabeling was carried out using methods similar to those described by others [31]. Briefly, synchronized L4 worms were fed ^35^S-methionine loaded OP50 bacteria for 4 h, washed and incubated with unlabeled OP50 for 1 h to purge radioactive intestinal bacteria, and then washed thoroughly with NGM buffer. Washed worms were flash frozen in liquid nitrogen and stored at −80°C before extraction. Protein was extracted from thawed samples by trichloroacetic acid-ethanol protein precipitation. Total protein concentration was measured by BCA assay (Pierce Biotechnology) and radioactivity incorporation by liquid scintillation counting.

### Temperature sensitive mutant phenotype assays

Synchronized temperature sensitive *let-60(ga89)* and *unc-15(el402)* mutant worms were grown on 51 mM NaCl NGM at the permissive temperature of 16°C. Adult worms were transferred to 300 mM NaCl plates at 16°C and allowed to lay eggs for 24 h. The number of eggs was counted, and those that did not develop past early larval stages were scored as larval arrest or defective egg hatching phenotypes.

### Measurement of worm protein levels

Single worms (50–70) were picked into 1 ml of NGM and incubated for 45 min to purge the intestine of bacteria. Purged worms were washed three times with buffer, resuspended in 100 µl of lysis buffer (0.1M NaOH, 0.2% SDS, 0.02% Tris HCl, pH 8.0), and incubated at 37°C for 4 h. Lysates were cleared by centrifugation and protein was measured by BCA assay.

### HB101 feeding experiments

Worms were fed either OP50 or HB101 *E. coli* strains to assess the effects of intracellular protein levels on Q35::YFP aggregation. HB101 fed worms developed ∼8–12 h more rapidly than worms fed OP50 bacteria. To ensure that Q35::YFP aggregation was quantified at identical developmental stages, measurements were performed in late L4 worms that had a fully developed vulva or in non-gravid young adults.

### RNAi experiments

RNA interference was performed by feeding worms a strain of *E. coli* engineered to transcribe dsRNA homologous to a target gene. The strains were obtained from commercially available RNAi feeding libraries (Geneservice Ltd, Cambridge, England; Open Biosystems, Huntsville, AL). A bacterial strain expressing 202 bases of dsRNA that are not homologous to any predicted *C. elegans* gene was used as a control for non-specific RNAi effects. dsRNA feeding was carried out for two days by transferring synchronized L1 larvae to agar plates seeded with control or specific RNAi bacteria.

### Statistical analysis

All data are presented as means ± S.E. Statistical significance was determined using Student's two-tailed *t* test. When comparing three or more groups, statistical significance was determined by one-way analysis of variance with a Tukey post test. P values of ≤0.05 were taken to indicate statistical significance.
